# Electric scooter–related accidents: a possible protective effect of helmet use on the head injury severity

**DOI:** 10.1007/s12024-022-00546-6

**Published:** 2022-11-04

**Authors:** Francesca Cittadini, Giovanni Aulino, Martina Petrucci, Silvia Valentini, Marcello Covino

**Affiliations:** 1 Department of Health Surveillance and Bioethics, Fondazione Policlinico A. Gemelli IRCCS, Università Cattolica del Sacro Cuore, Largo F. Vito, 00168 Rome, Italy; 2 Emergency Medicine Department, Fondazione Policlinico Universitario Agostino Gemelli-IRCCS, Università Cattolica del Sacro Cuore Di Roma, Largo A. Gemelli 8, 00168 Rome, Italy

**Keywords:** Electric scooter accidents, E-scooter, Injury patterns, Emergency Department, Helmets

## Abstract

Despite electric scooter use has proliferated in Italy since 2019, actionable data regarding injury incidence and patterns associated with electric scooter accidents are limited. This study aims at analyzing the rate, clinical, and demographic features of electric scooter accidents accessed to the Emergency Department (ED) of Fondazione Policlinico Universitario A. Gemelli IRCSS (Rome, Italy). This retrospective study included all patients older than 18 years riding an electric scooter in the ED from June 2019 to April 2022. Personal data, injury circumstances, helmet use, and health data were collected. Abbreviated Injury Scale (AIS) codes of all diagnoses were recorded, and the Injury Severity Score (ISS) was calculated for each patient. The analysis includes 92 patients admitted to the ED due to an e-scooter accident during the study period, with an increase in years. Thirty-two patients presented bone fractures especially concerning the extremities and the face districts. The median Injury Severity Score in the study cohort was 3, with the highest AIS represented by AIS Pelvic-Extremity and AIS External. Moreover, statistical significance was found between AIS Head-Neck and severity of trauma. E-scooters have become a familiar sight in cities worldwide recently, with many new companies renting them for use. But their arrival has also brought new safety concerns. Although most injuries reported are minor, the meager rate of helmet use is critical. Implementing compulsory helmet use for electric scooters for all ages could be a protective factor for being patient with head trauma on urban streets.

## Introduction

At the end of 2017, rental electric scooters (e-scooters) were first introduced in the USA as a new means of transport [1]. In Italy, approval for using E-scooters in public road traffic was granted on June 5, 2019. Since then, e-scooters have been distributed throughout large cities, such as Rome and Milan, primarily via rental companies. Subsequently, they became a prevalent means of private transport, thanks partly to government incentives to reduce environmental pollution. In Italy, the maximum speed of the e-scooter is limited to 20 km/h, the rated power of the electric motor may not exceed 500 watts, and it must have an acoustic beacon. In addition, use is permitted from a minimum age of 14 years, exclusively with a specific driving license for mopeds [2].

Although Italian legislation regarding scooters has been implemented, helmets are only compulsory under the age of 18 [3].

While there are warnings from companies regarding using these e-scooters with a maximum speed of 20 km/h, injuries due to e-scooter accidents have been reported in many countries because of the insufficient infrastructure in the cities [4]. In Italy, deaths have already been reported following e-scooter accidents [5]. Through the analysis of e-scooter accidents, the effects of e-scooters on public health will be elucidated, and legal regulations regarding the use of e-scooters will gain importance.

This study aims at analyzing the rate, the clinical, and the demographic features of the e-scooter accidents accessed to the Emergency Department (ED) of Fondazione Policlinico Universitario A. Gemelli IRCSS (Rome, Italy).

## Materials and methods

### Data collection

This retrospective study included all patients older than 18 years riding an electric scooter. Data collection took place at ED of Fondazione Policlinico Universitario A. Gemelli IRCSS (Rome, Italy) from June 2019 to April 2022. The ED is based in a University teaching hospital, with an annual attendance of about 75,000 patients, 20% for trauma. The ED is also a major trauma center.

Patient data were analyzed for patient characteristics (gender, age, nationality, injury circumstances, helmet use and type of injury, emergency room access code, and date and time of admission).

Final diagnoses of each patient (10th.version of the International Classification of Diseases and radiology reports) were documented [6].

Abbreviated Injury Scale (AIS) codes (ver. 2005) of all diagnoses were recorded, and the Injury Severity Score (ISS) was calculated for each patient [7].

The injury patterns were grouped into severe trauma and non-severe trauma for a detailed analysis of the injury patterns. Severe trauma was defined as cases that presented an ISS greater or equal than 16. Non-severe trauma was defined as injuries that gave an ISS less than 16.

### Statistical analysis

Categorical variables are presented as numbers and percentages and statistically compared at univariate analysis by chi-square test, with Yates correction or Fisher’s exact test if appropriate. Continuous variables are presented as median [interquartile range] and compared by the Mann–Whitney *U* test. The significance level was set at 0.05, two-sided. All data were analyzed by SPSS V25® (IBM, Armonk, NY, USA).

## Results

Ninety-two patients were admitted to the ED due to an e-scooter accident during the study period.

**Fig. 1:**
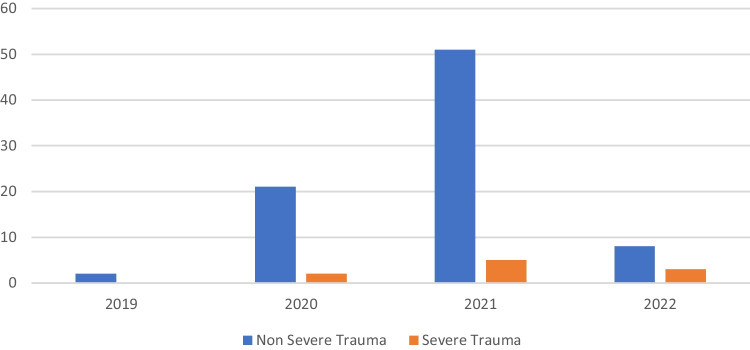
Breakdown of e-scooter accidents by year

Regarding the year distribution, there was an increase in cases (two in 2019, twenty-three in 2020, fifty-six in 2021, and eleven in the first four months of 2022, respectively) (Fig. 1).

The median age of the patients was 28 [IQR, 19.5–39], with 16 (17.4%) female patients. About these thirty-six were admitted with the code “emergency” or “urgency” (twenty-three and thirteen, respectively) (Table 1).

**Table 1:** Characteristics of patients admitted to the emergency department by access code

	**All patients** ***n* 92**	**1 Emergency** ***n* 23**	**2 Urgency** ***n* 13**	**3 Ambulatory** ***n* 56**	***P* value**
Age	28 [19.5–39]	28 [23–39]	30 [19.5–39.5]	28 [18–38.5]	0.945
Sex (male)	76 (82.6%)	18 (78.3%)	11 (84.6%)	47 (83.9%)	0.816
ED access on nightshift (*12 A.M. to 8 A.M.*)	28 (30.4%)	3 (13.0%)	8 (61.5%)	16 (28.6%)	**0.010**
ED access on weekends (*Saturday, Sunday*)	29 (31.5%)	3 (13.0%)	8 (61.5%)	17 (30.4%)	0.127
ED access by Emergency Service	40 (43.5%)	20 (87.0%)	7 (53.8%)	13 (23.2%)	** < 0.001**
***Trauma severity***					
ISS	3 [1–5]	5 [1–17]	3 [1–5]	2.5 [1–4]	0.126
AIS head neck	0 [0–0]	0 [0–1]	0 [0–1]	0 [0–0]	**0.038**
AIS face	0 [0–0]	0 [0–0]	0 [0–0]	0 [0–0.75]	0.905
AIS chest	0 [0–0]	0 [0–0]	0 [0–0]	0 [0–0]	0.949
AIS abdomen	0 [0–0]	0 [0–0]	0 [0–0]	0 [0–0]	1.000
AIS pelvi-extremity	1 [0–2]	0 [0–2]	0 [0–1.5]	1 [0–2]	0.616
AIS external	1 [0–1]	1 [0–1]	0 [0.50–1]	1 [0–1]	0.424
***Outcomes***					
Bone fracture	32 (34.8%)	10 (43.5%)	5 (38.5%)	17 (30.4%)	0.515
Skin wound (*suture needed*)	16 (17.4%)	2 (8.7%)	3 (32.1%)	11 (19.6%)	0.427
Concussion	24 (26.1%)	6 (26.1%)	6 (46.2%)	12 (21.4%)	0.188
Need for hospital admission > 24 h	24 (26.1%)	13 (56.5%)	5 (38.5%)	6 (10.7%)	** < 0.001**

Statistically significant results are highlighted in bold (< 0.05)

Twenty-eight patients (30.4%) were admitted during the night (between 12 P.M. and 8 A.M.). Twenty-nine accidents (31.5%) occurred during the weekend (between Saturday 0 A.M. and Sunday noon). The medical history shows that only one patient wore a helmet. In most cases (94.6%), the injury was caused by either a fall or loss of balance; in three cases, there was a collision with a car, and two patients were not documented.

Thirty-two patients (34.8%) presented bone fractures, and sixteen (17.4%) needed sutures for skin wounds. Only two patients had multiple fractures (Table 1).

Among patients who had fractures, the most frequently affected region is that of the extremities (50%), followed by the facial Section (31.25%) (Table 2).

**Table 2:** Breakdown of patients who presented with fractures according to injury context, helmet use and fractures type

	***Non-severe trauma (total 26)***	***Severe trauma (total 6)***
***Injury context***		
Self-inflicted accident Motor vehicle involvement	22 (84.6%) 4 (15.4%)	5 (83.3%) 1 (16.7%)
***Helmet use***	1 (3.8%)	0 (0%)
***Fractures***		
Single fracture Multiple fracture	26 (100%) 0 (0%)	4 (66.7%) 2 (33.3%)
**Face**	**7 (26.9%)**	**3 (50%)**
Midface	7 (100%)	3 (100%)
**Neck**	**1 (3.8%)**	**0 (0%)**
Thyroid cartilage	1 (100%)	0 (0%)
**Chest (total)**	**1 (3.8%)**	**5 (83.3%)**
Clavicle Ribs	1 (100%) 0 (0%)	1 (20%) 4 (80%)
**Upper extremity (total)**	**11 (42.30%)**	**1 (16.7%)**
Radius Ulna Hand	3 (27.3%) 1 (9.1%) 7 (63.6%)	1 (100%) 0 (0%) 0 (0%)
**Lower extremity (total)**	**2 (7.7%)**	**3 (50%)**
Femur Tibia Fibula	1 (50%) 0 (0%) 1 (50%)	1 (33.3%) 1 (33.3%) 1 (33.3%)

Finally, twenty-four (26.1%) presented concussion, and twenty-four (26.1%) needed a hospital admission lasting more than 24 h (Table 1).

The median Injury Severity Score in the study cohort was 3 [IQR, 1–5]. Regarding the injured regions involved in trauma, the body region with the highest AIS was Pelvic-Extremity with a median AIS of 1 [IQR, 0–2] and the External with a median AIS of 1 [IQR, 0–1] (Table 1).

Moreover, statistical significance was found between head-neck AIS and severity of trauma.

Then, based on the ISS, the entire cohort was divided into two groups: non-severe trauma with an ISS < 16 and severe trauma with ISS ≥ 16 (Table 3).

**Table 3:** Breakdown of the entire cohort by ISS into non-severe trauma and severe trauma

	**Non-severe trauma (ISS < 16)** ***n* 82**	**Severe trauma (ISS ≥ 16)** ***n* 10**	***P* value**
Age	30 [20.5–39.25]	23.50 [18.5–26]	0.105
Sex (male)	69 (84.1%)	7 (70%)	0.370
ED access on nightshift *(12 P.M. to 8 A.M.)*	25 (30.5%)	3 (30%)	1.000
ED access on weekends *(Saturday, Sunday)*	27 (32.9%)	2 (20%)	0.496
ED access by emergency service	34 (41.5%)	6 (60%)	0.321
***Trauma severity***			
ISS	2 [1–4.25]	17.5 [16.75–26]	** < 0.001**
AIS head neck	0 [0–0]	0 [0–4]	**0.034**
AIS face	0 [0–0]	0 [0–4]	0.076
AIS chest	0 [0–0]	0 [0–0]	0.198
AIS abdomen	0 [0–0]	0 [0–0]	1.000
AIS pelvi-extremity	1 [0–2]	0.5 [0–4.25]	0.463
AIS external	1 [0–1]	1 [0.75–1]	0.192
***Outcomes***			
Bone fracture	26 (31.7%)	6 (60%)	0.091
Skin wound (*suture needed*)	16 (19.5%)	0	0.200
Concussion	22 (26.8%)	2 (20%)	1.000
Need for hospital admission > 24 h	17 (20.1)%	10 (100%)	** < 0.001**

Statistically significant results are highlighted in bold (< 0.05)

### Non-severe trauma

The 82 patients admitted to the ED presented an ISS < 16 with a median ISS of 2 [IQR, 1–4.25] had a median age of 30 [IQR, 20.5–39.25]; 30 (15.9%) were female.

Among this group, 26 (31.7%) presented bone fractures, and 16 (19.5%) needed a suture for a skin wound. Only a minority (20.1%) required hospital admission longer than 24 h (Table 3).

### Severe trauma

Only ten patients admitted to the ED presented an ISS greater or equal to 16 with a median ISS of 17.5 [IQR, 16.75–26] and had a median age of 23.50 [IQR 18.5–26]; three patients (30%) were female. The majority (60%) accessed the ED by Emergency Service.

Of these, the majority (60%) presented bone fractures, and all the patients required Hospital Admission greater than 24 h (Table 3).

## Discussion

This retrospective study analyzed the injury pattern following accidents with e-scooter who were admitted at the ED of Fondazione Policlinico Universitario A. Gemelli IRCSS (Rome, Italy) from June 2019 to April 2022.

Due to the increasing use of e-scooter, studies focusing on injury patterns are increasing in European countries, although this topic has never been analyzed in Italy [8–10].

In our cohort, ninety-two patients were admitted to the ED due to an e-scooter accident during the study period. The median age was 28, and most patients were male (82.6%). These data confirmed that males are more frequently involved in e-scooter accidents and that younger age groups were more commonly involved in accidents than older age groups [11–13].

Moreover, as Stigson et al. have shown, the injuries by e-scooter have increased in Italy over the years [14].

These data could be due to the increasing use of these means of transportation on the one hand and fewer restrictive measures due to the coronavirus pandemic in 2021 compared with 2020, on the other [15, 16].

In addition, a statistical correlation was observed with ED access during the night shift. This figure could be since these vehicles are hardly visible at night despite the requirement to wear the reflective vest or high-visibility retro-reflective suspenders [3]. Instead, regarding the dynamics, as with other studies, ours shows that the primary mechanism of damage is the fall [17].

In most of the patients included in the study, as reported by the low rate of fractures and the low rate of ISS and AIS injury scores, the reported injuries were minor, which could be related to the speed limits imposed for this type of vehicle [18].

The most frequently affected region was the extremities, especially the upper limbs like that observed in specific non-motorized mobility devices, which present a delicate balance [19]. Among upper limb fractures, the most commonly affected region was the hand: this could be mainly accounted for by the fact that these vehicles are also used on urban roads that may have a road surface that has potholes that can easily alter the static of the car and cause the fall. For these reasons, these modes of transportation should only run on dedicated streets such as bike lanes and not on urban roads.

However, although the injuries are minor, as reported in other studies, another critical element of the study is the meager rate of helmet use [20–22].

In addition, in our study, the correlation between AIS head neck and trauma severity was statistically significant, so that that helmet use could reduce trauma severity.

However, despite increased restrictive measures against these vehicles in Italy, helmets are still not compulsory for individuals over 18 [3].

Although the Italian parliament has not introduced this measure, some cities such as Florence have made helmets mandatory, so Latium could also adopt this measure to reduce the severity of injuries [23].

The strengths and weaknesses of this study must be taken into account. Because enrollment is restricted to a single ED, it is possible that it may not accurately represent the characteristic of injuries in Italy. However, this study is the first to examine the aspects of injuries and the clinical outcome of the patients admitted for an e-scooter accident in Italy.

Thus, our patient population may be skewed towards more severe injuries that warrant multiple providers’ patient interviews and documentation. Secondly, before the injury, abstraction of helmet use relied on medical provider query of helmet use and subsequent medical record documentation. This may have led to the underrepresentation of helmet use within the present cohort. On the other hand, incorporating patients’ self-reported helmet use portends the risk of overrepresentation. Overall, the risk of over or under-representation of helmet use was likely minimized as the currently reported rates are similar to prior observational studies. Finally, as a retrospective review, this study does not encompass long-term outcomes of consequences secondary to e-scooter injuries. Nonetheless, this review reports a valuable investigation of modifiable risk factors for future public health advocacy of safety measures that minimize morbid e-scooter injuries.

As e-scooters flood the shared micro-mobility industry with minimal public safety regulations, the incidence of both minor and significant e-scooter injuries is likely to continue to rise. Low rates of helmet use among electric scooter users could be linked to the high prevalence of head injuries following electric scooter–associated trauma. Hospital admission would benefit from mandatory helmet use in urban streets, reduced speed limits, and dedicated lanes. Future investigations may provide further insight into effective preventative public health measures to decrease the risk of morbid e-scooter injuries and to determine the contribution of alcohol or drug consumption to the mechanism of injuries of these means of transport.

## Key points

1. Although wearing a reflective vest or high-visibility retroreflective harness is required, a statistical association was found between access to the ED during the night shift. This is possibly because these vehicles are not very visible at night.
2. According to the low rate of fractures, low rate of ISS and AIS injury ratings, and the majority of patients included in the study, the reported injuries were typically minor, which may be related to the speed limitations set for this type of vehicle.
3. Since there is a statistically significant relationship between head-neck AIS and trauma intensity, wearing a helmet, even though it is not currently required for all ages, may lessen the degree of trauma.

## Data Availability

My manuscript has associated data in a data repository.
